# Supplementary material for: Abnormal p53 High-Grade Endometrioid Endometrial Cancer: A Systematic Review and Meta-Analysis
Source: Cancers (Basel). 2024 Dec 26;17(1):38. doi: 10.3390/cancers17010038 (PMC11718986; doi:10.3390/cancers17010038)
Supplement: Supplementary file 1 [file cancers-17-00038-s001.zip › cancers-3318691-supplementary.pdf]

Supplementary Material

Table S1. Preferred Reporting Items for Systematic Reviews and Meta-analyses checklist.

Section and Topic	Item #	Checklist item	Location where item is reported
**TITLE**			
Title	1	Identify the report as a systematic review.	Page 1
**ABSTRACT**			
Abstract	2	See the PRISMA 2020 for Abstracts checklist.	Page 4
**INTRODUCTION**			
Rationale	3	Describe the rationale for the review in the context of existing knowledge.	Pages 5-7
Objectives	4	Provide an explicit statement of the objective(s) or question(s) the review addresses.	Pages 6-7
**METHODS**			
Eligibility criteria	5	Specify the inclusion and exclusion criteria for the review and how studies were grouped for the syntheses.	Pages 7-8
Information sources	6	Specify all databases, registers, websites, organizations, reference lists and other sources searched or consulted to identify studies. Specify the date when each source was last searched or consulted.	Pages 8-9
Search strategy	7	Present the full search strategies for all databases, registers and websites, including any filters and limits used.	Pages 8-9<br>Appendix B
Selection process	8	Specify the methods used to decide whether a study met the inclusion criteria of the review, including how many reviewers screened each record and each report retrieved, whether they worked independently, and if applicable, details of automation tools used in the process.	Page 9
Data collection process	9	Specify the methods used to collect data from reports, including how many reviewers collected data from each report, whether they worked independently, any processes for obtaining or confirming data from study investigators, and if applicable, details of automation tools used in the process.	Page 9
Data items	10a	List and define all outcomes for which data were sought. Specify whether all results that were compatible with each outcome domain in each study were sought (e.g. for all measures, time points, analyses), and if not, the methods used to decide which results to collect.	Page 10
	10b	List and define all other variables for which data were sought (e.g. participant and intervention characteristics, funding sources). Describe any assumptions made about any missing or unclear information.	Pages 9-10
Study risk of bias assessment	11	Specify the methods used to assess risk of bias in the included studies, including details of the tool(s) used, how many reviewers assessed each study and whether they worked independently, and if applicable, details of automation tools used in the process.	Page 10

Section and Topic	Item #	Checklist item	Location where item is reported
Effect measures	12	Specify for each outcome the effect measure(s) (e.g. risk ratio, mean difference) used in the synthesis or presentation of results.	Pages 10-11
Synthesis methods	13a	Describe the processes used to decide which studies were eligible for each synthesis (e.g. tabulating the study intervention characteristics and comparing against the planned groups for each synthesis (item #5)).	Page 10
	13b	Describe any methods required to prepare the data for presentation or synthesis, such as handling of missing summary statistics, or data conversions.	Pages 10-11
	13c	Describe any methods used to tabulate or visually display results of individual studies and syntheses.	Pages 10-12
	13d	Describe any methods used to synthesize results and provide a rationale for the choice(s). If meta-analysis was performed, describe the model(s), method(s) to identify the presence and extent of statistical heterogeneity, and software package(s) used.	Pages 10-12
	13e	Describe any methods used to explore possible causes of heterogeneity among study results (e.g. subgroup analysis, meta-regression).	Pages 11-12
	13f	Describe any sensitivity analyses conducted to assess robustness of the synthesized results.	Page 11
Reporting bias assessment	14	Describe any methods used to assess risk of bias due to missing results in a synthesis (arising from reporting biases).	Page 12
Certainty assessment	15	Describe any methods used to assess certainty (or confidence) in the body of evidence for an outcome.	N/A
**RESULTS**			
Study selection	16a	Describe the results of the search and selection process, from the number of records identified in the search to the number of studies included in the review, ideally using a flow diagram.	Pages 12-13<br>Figure 1
	16b	Cite studies that might appear to meet the inclusion criteria, but which were excluded, and explain why they were excluded.	N/A
Study characteristics	17	Cite each included study and present its characteristics.	Pages 13-18<br>Table 1
Risk of bias in studies	18	Present assessments of risk of bias for each included study.	Pages 19-22<br>Table 2
Results of individual studies	19	For all outcomes, present, for each study: (a) summary statistics for each group (where appropriate) and (b) an effect estimate and its precision (e.g. confidence/credible interval), ideally using structured tables or plots.	Table 1<br>Figures 2,

Section and Topic	Item #	Checklist item	Location where item is reported
			3, and 4
Results of syntheses	20a	For each synthesis, briefly summarize the characteristics and risk of bias among contributing studies.	N/A
	20b	Present results of all statistical syntheses conducted. If meta-analysis was done, present for each the summary estimate and its precision (e.g. confidence/credible interval) and measures of statistical heterogeneity. If comparing groups, describe the direction of the effect.	Pages 23-28<br>Figures 2, 3, and 4
	20c	Present results of all investigations of possible causes of heterogeneity among study results.	Page 27<br>Figure 5<br>Appendix F
	20d	Present results of all sensitivity analyses conducted to assess the robustness of the synthesized results.	Pages 23-24<br>Appendix C
Reporting biases	21	Present assessments of risk of bias due to missing results (arising from reporting biases) for each synthesis assessed.	Pages 27-28<br>Appendix D and E
Certainty of evidence	22	Present assessments of certainty (or confidence) in the body of evidence for each outcome assessed.	N/A
**DISCUSSION**			
Discussion	23a	Provide a general interpretation of the results in the context of other evidence.	Pages 28-30
	23b	Discuss any limitations of the evidence included in the review.	Pages 30-31
	23c	Discuss any limitations of the review processes used.	N/A
	23d	Discuss implications of the results for practice, policy, and future research.	Pages 28-29; 31
**OTHER INFORMATION**			
Registration and protocol	24a	Provide registration information for the review, including register name and registration number, or state that the review was not registered.	Page 7
	24b	Indicate where the review protocol can be accessed, or state that a protocol was not prepared.	Page 7
	24c	Describe and explain any amendments to information provided at registration or in the protocol.	N/A

Section and Topic	Item #	Checklist item	Location where item is reported
Support	25	Describe sources of financial or non-financial support for the review, and the role of the funders or sponsors in the review.	Page 33
Competing interests	26	Declare any competing interests of review authors.	Page 34
Availability of data, code and other materials	27	Report which of the following are publicly available and where they can be found: template data collection forms; data extracted from included studies; data used for all analyses; analytic code; any other materials used in the review.	Page 33

N/A, not applicable.

From: Page MJ, McKenzie JE, Bossuyt PM, Boutron I, Hoffmann TC, Mulrow CD, et al. The PRISMA 2020 statement: an updated guideline for reporting systematic reviews. BMJ 2021;372:n71. doi: 10.1136/bmj.n71

Table S2. Search strategy in PubMed, EMBASE, Cochrane Library, Scopus, and Web of Science.

Query	Search
**PubMed**	*ALL FIELDS*
#1	"high-grade endometrioid endometrial cancer*" OR HGEEC OR "high grade endometrioid endometrial cancer*" OR "high-grade endometrioid endometrial carcinoma*" OR "high grade endometrioid endometrial carcinoma*" OR "G3 endometrioid endometrial cancer*" OR "G3 endometrioid endometrial carcinoma*" OR "G3 endometrial carcinoma*" OR "grade 3 endometrioid endometrial cancer*" OR "grade 3 endometrioid endometrial carcinoma*" OR "grade 3 endometrial carcinoma*" OR "endometrial cancer*" OR "endometrial carcinoma*" OR EC
#2	"p53 mutation*" OR "p53 mutated" OR "p53 abnormal" OR "p53 positive*" OR "p53 positivity" OR "p53 immunohistochemistry" OR "TP53 mutation"
#3	#1 AND #2
**EMBASE**	*Title/Abstract/Author Supplied Keywords*
#1	"high-grade endometrioid endometrial cancer*" OR HGEEC OR "high grade endometrioid endometrial cancer*" OR "high-grade endometrioid endometrial carcinoma*" OR "high grade endometrioid endometrial carcinoma*" OR "G3 endometrioid endometrial cancer*" OR "G3 endometrioid endometrial carcinoma*" OR "G3 endometrial carcinoma*" OR "grade 3 endometrioid endometrial cancer*" OR "grade 3 endometrioid endometrial carcinoma*" OR "grade 3 endometrial carcinoma*" OR "endometrial cancer*" OR "endometrial carcinoma*" OR EC
#2	"p53 mutation*" OR "p53 mutated" OR "p53 abnormal" OR "p53 positive*" OR "p53 positivity" OR "p53 immunohistochemistry" OR "TP53 mutation"
#3	#1 AND #2
**Cochrane Library**	*Title, abstract, keyword*
#1	"high-grade endometrioid endometrial cancer*" OR HGEEC OR "high grade endometrioid endometrial cancer*" OR "high-grade endometrioid endometrial carcinoma*" OR "high grade endometrioid endometrial carcinoma*" OR "G3 endometrioid endometrial cancer*" OR "G3 endometrioid endometrial carcinoma*" OR "G3 endometrial carcinoma*" OR "grade 3 endometrioid endometrial cancer*" OR "grade 3 endometrioid endometrial carcinoma*" OR "grade 3 endometrial carcinoma*" OR "endometrial cancer*" OR "endometrial carcinoma*" OR EC
#2	"p53 mutation*" OR "p53 mutated" OR "p53 abnormal" OR "p53 positive*" OR "p53 positivity" OR "p53 immunohistochemistry" OR "TP53 mutation"
#3	#1 AND #2

Scopus	*Article title, abstract, keywords*
#1	"high-grade endometrioid endometrial cancer*" OR HGEEC OR "high grade endometrioid endometrial cancer*" OR "high-grade endometrioid endometrial carcinoma*" OR "high grade endometrioid endometrial carcinoma*" OR "G3 endometrioid endometrial cancer*" OR "G3 endometrioid endometrial carcinoma*" OR "G3 endometrial carcinoma*" OR "grade 3 endometrioid endometrial cancer*" OR "grade 3 endometrioid endometrial carcinoma*" OR "grade 3 endometrial carcinoma*" OR "endometrial cancer*" OR "endometrial carcinoma*" OR EC
#2	"p53 mutation*" OR "p53 mutated" OR "p53 abnormal" OR "p53 positive*" OR "p53 positivity" OR "p53 immunohistochemistry" OR "TP53 mutation"
#3	#1 AND #2
**Web of Science**	*TOPIC - title, abstract, author keywords, and Keywords Plus*
#1	"high-grade endometrioid endometrial cancer*" OR HGEEC OR "high grade endometrioid endometrial cancer*" OR "high-grade endometrioid endometrial carcinoma*" OR "high grade endometrioid endometrial carcinoma*" OR "G3 endometrioid endometrial cancer*" OR "G3 endometrioid endometrial carcinoma*" OR "G3 endometrial carcinoma*" OR "grade 3 endometrioid endometrial cancer*" OR "grade 3 endometrioid endometrial carcinoma*" OR "grade 3 endometrial carcinoma*" OR "endometrial cancer*" OR "endometrial carcinoma*" OR EC
#2	"p53 mutation*" OR "p53 mutated" OR "p53 abnormal" OR "p53 positive*" OR "p53 positivity" OR "p53 immunohistochemistry" OR "TP53 mutation"
#3	#1 AND #2

Figure S1. Sensitivity analysis for overall survival of patients with p53-abnormal G3 endometrioid endometrial carcinomas.

CI, confidence interval; HR, hazard ratio.

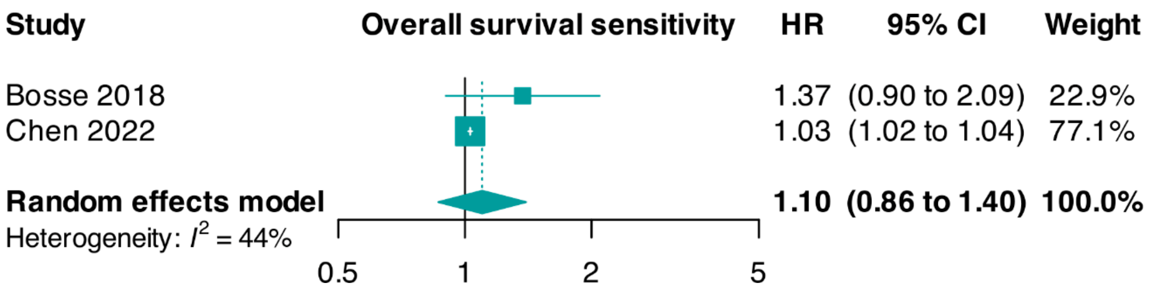

Figure S2. Sensitivity analysis for progression-free survival of patients with p53-abnormal G3 endometrioid endometrial carcinomas.

CI, confidence interval; HR, hazard ratio.

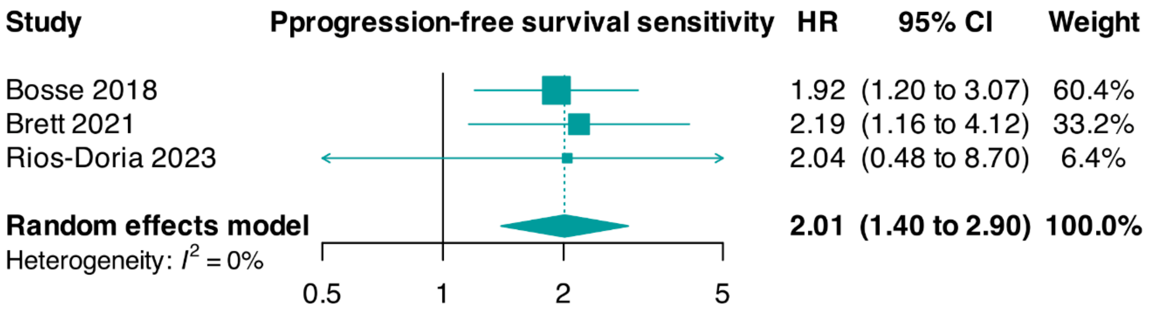

Figure S3. p-curve analysis of abnormal p53 pooled prevalence estimate. CI, confidence interval.

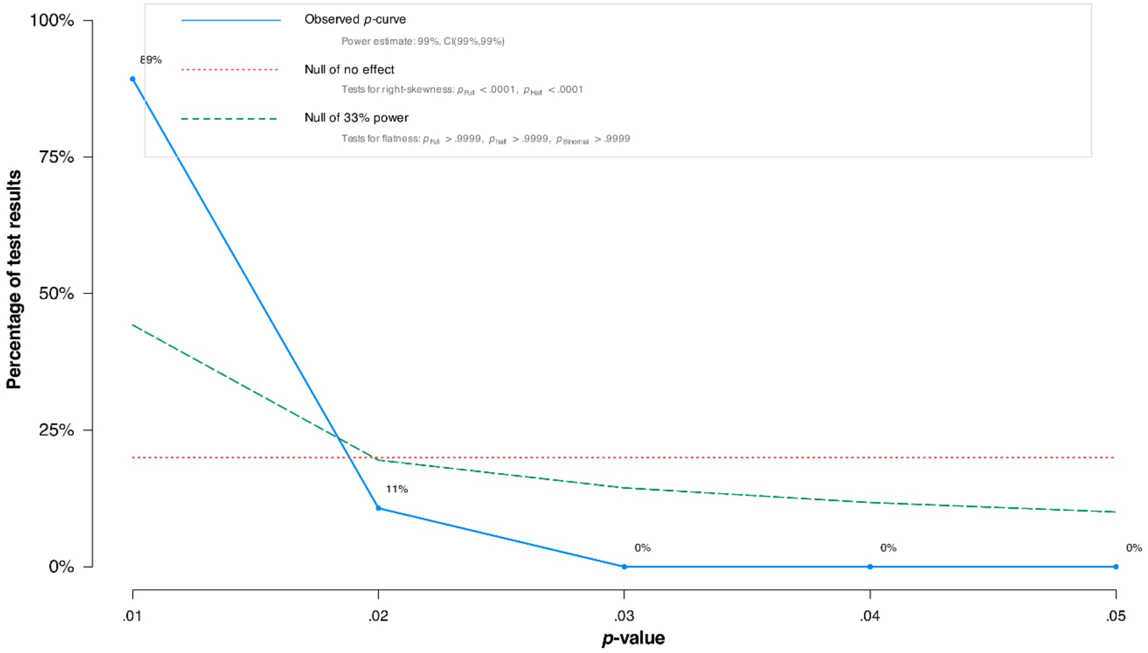

Note: The observed *p*-curve includes 56 statistically significant ($p < .05$) results, of which 56 are $p < .025$.
There was one additional result entered but excluded from *p*-curve because it was $p > .05$.

Figure S4. Trim-and-fill analysis of abnormal p53 pooled prevalence.

CI, confidence interval.

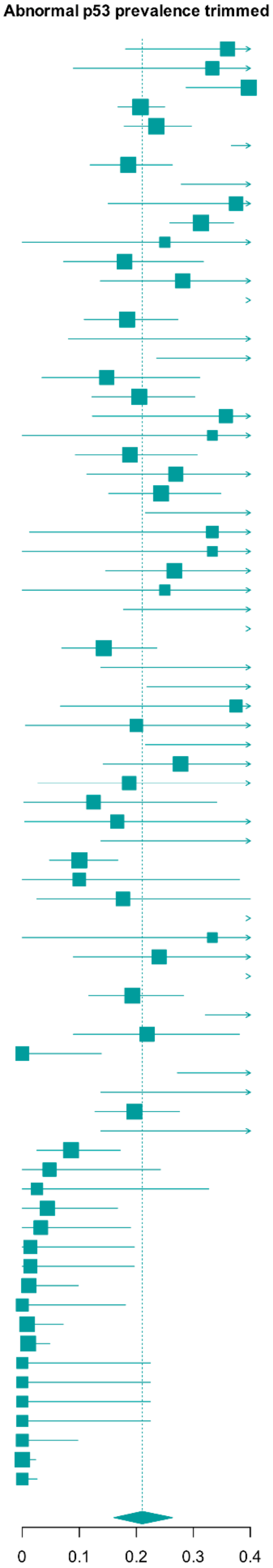

Study	Proportion	95% CI	Weight
Alvarez 2012	0.36	(0.18 to 0.56)	1.5%
Balestra 2023	0.33	(0.09 to 0.63)	1.3%
Betella 2022	0.40	(0.29 to 0.51)	1.7%
Bosse 2018	0.21	(0.17 to 0.25)	1.8%
Brett 2021	0.23	(0.18 to 0.30)	1.8%
Buchynska 2017	0.55	(0.37 to 0.73)	1.5%
Leon-Castillo 2020 b	0.19	(0.12 to 0.26)	1.7%
Chen 2022	0.40	(0.28 to 0.53)	1.7%
Cuevas 2019	0.38	(0.15 to 0.63)	1.4%
Dai 2021	0.31	(0.26 to 0.37)	1.8%
Daniilidou 2013	0.25	(0.00 to 0.79)	0.8%
Dankai 2023	0.18	(0.07 to 0.32)	1.6%
Devereaux 2022	0.28	(0.14 to 0.45)	1.5%
Dobrzycka 2024	0.58	(0.42 to 0.73)	1.6%
Rios-Doria 2023	0.18	(0.11 to 0.27)	1.7%
Enomoto 1995	0.43	(0.08 to 0.81)	1.0%
Feng 2005	0.47	(0.24 to 0.71)	1.4%
Hoang 2017	0.15	(0.03 to 0.31)	1.5%
Huvila 2023	0.21	(0.12 to 0.30)	1.7%
Inaba 2005	0.36	(0.12 to 0.63)	1.3%
Jones 1997	0.33	(0.00 to 0.94)	0.7%
JuttaHuvila 2018	0.19	(0.09 to 0.31)	1.6%
Kang 2022	0.27	(0.11 to 0.46)	1.5%
Kato 2022	0.24	(0.15 to 0.35)	1.7%
Kihana 1995	0.50	(0.22 to 0.78)	1.3%
Kitazono 2023	0.33	(0.01 to 0.76)	1.0%
Kobayashi 2021	0.33	(0.00 to 0.94)	0.7%
Kogata 2018	0.27	(0.15 to 0.41)	1.6%
Koshiyama 1993	0.25	(0.00 to 0.79)	0.8%
Lax 2000	0.43	(0.18 to 0.70)	1.3%
Leon-Castillo 2020 a	0.59	(0.48 to 0.69)	1.7%
Li 2022	0.14	(0.07 to 0.24)	1.7%
Li 1996	0.60	(0.14 to 0.98)	0.9%
Maeda 2002	0.56	(0.22 to 0.87)	1.1%
Massenkeil 1995	0.37	(0.07 to 0.74)	1.1%
Matsumoto 2023	0.20	(0.01 to 0.51)	1.2%
Mazurek 2008	0.50	(0.22 to 0.78)	1.3%
Moreira 2023	0.28	(0.14 to 0.44)	1.6%
Nostrand 1998	0.19	(0.03 to 0.42)	1.4%
Nout 2012	0.12	(0.00 to 0.34)	1.4%
Oberndorfer 2021	0.17	(0.00 to 0.44)	1.3%
Okamoto 1991	0.60	(0.14 to 0.98)	0.9%
Pain 2023	0.10	(0.05 to 0.17)	1.7%
Da Cruz Paula 2021	0.10	(0.00 to 0.38)	1.2%
Pijnenborg 2006	0.18	(0.03 to 0.40)	1.4%
Riethdorf 1996	0.69	(0.52 to 0.83)	1.6%
Sakuragi 2001	0.33	(0.00 to 0.94)	0.7%
Sakuragi 2005	0.24	(0.09 to 0.43)	1.5%
Sawairiitoh 1994	0.83	(0.41 to 1.00)	1.0%
Schultheis 2016	0.19	(0.12 to 0.28)	1.7%
Shiozawa 1997	0.67	(0.32 to 0.94)	1.1%
Singh 2020	0.22	(0.09 to 0.38)	1.5%
Timmerman 2020	0.00	(0.00 to 0.14)	1.3%
Tresa 2022	0.46	(0.27 to 0.66)	1.5%
Tsuda and Hirohashi 1992	0.60	(0.14 to 0.98)	0.9%
Vermij 2022	0.20	(0.13 to 0.28)	1.7%
Yamauchi 1996	0.60	(0.14 to 0.98)	0.9%
Filled: Chen 2022	0.09	(0.03 to 0.17)	1.7%
Filled: Lax 2000	0.05	(0.00 to 0.24)	1.3%
Filled: Enomoto 1995	0.03	(0.00 to 0.33)	1.0%
Filled: Tresa 2022	0.04	(0.00 to 0.17)	1.5%
Filled: Feng 2005	0.03	(0.00 to 0.19)	1.4%
Filled: Kihana 1995	0.01	(0.00 to 0.20)	1.3%
Filled: Mazurek 2008	0.01	(0.00 to 0.20)	1.3%
Filled: Buchynska 2017	0.01	(0.00 to 0.10)	1.5%
Filled: Maeda 2002	0.00	(0.00 to 0.18)	1.1%
Filled: Dobrzycka 2024	0.01	(0.00 to 0.07)	1.6%
Filled: Leon-Castillo 2020 a	0.01	(0.00 to 0.05)	1.7%
Filled: Li 1996	0.00	(0.00 to 0.22)	0.9%
Filled: Okamoto 1991	0.00	(0.00 to 0.22)	0.9%
Filled: Tsuda and Hirohashi 1992	0.00	(0.00 to 0.22)	0.9%
Filled: Yamauchi 1996	0.00	(0.00 to 0.22)	0.9%
Filled: Shiozawa 1997	0.00	(0.00 to 0.10)	1.1%
Filled: Riethdorf 1996	0.00	(0.00 to 0.02)	1.6%
Filled: Sawairiitoh 1994	0.00	(0.00 to 0.03)	1.0%
**Random effects model**	**0.21**	**(0.16 to 0.26)**	**100.0%**

Heterogeneity: $I^2 = 82\%$, $\tau^2 = 0.05$

Table S3. Results of meta regression analysis of the pooled prevalence of abnormal p53.

Regressor	Studies	Tau^2	I^2	R^2	p-value	Estimate	LB 95% CI	UB 95% CI
Year of publication	57.00	0.01	0.68	0.36	<0.0001	-0.01	-0.01	-0.01
Patient age (median)	31.00	0.02	0.82	0.02	0.23	-0.01	-0.02	0.00
FIGO stage III-IV	25.00	0.02	0.81	0.00	0.61	-0.09	-0.50	0.31
Overall risk of bias	57.00	0.02	0.76	0.00	0.31	-0.06	-0.18	0.06

CI, confidence interval; FIGO, International Federation of Obstetrics and Gynecology; LB, lower bound; UB, upper bound.
